# Low-carbohydrate, high-protein diet score and risk of incident cancer; a prospective cohort study

**DOI:** 10.1186/1475-2891-12-58

**Published:** 2013-05-07

**Authors:** Lena Maria Nilsson, Anna Winkvist, Ingegerd Johansson, Bernt Lindahl, Göran Hallmans, Per Lenner, Bethany Van Guelpen

**Affiliations:** 1 Department of Public Health and Clinical Medicine,Nutritional Research, Umeå University, Umeå SE-90185, Sweden; 2 Department of Internal Medicine and Clinical Nutrition, Sahlgrenska Academy, University of Gothenburg, Göteborg SE-40530, Sweden; 3 Department of Odontology, Umeå University, Umeå SE-90185, Sweden; 4 Department of Public Health and Clinical Medicine, Umeå University, Umeå SE-90185, Sweden; 5 Department of Oncology and Radiation Sciences, Oncological Center, Umeå University, Umeå SE-90185, Sweden; 6 Department of Medical Biosciences, Pathology, Umeå University, Umeå SE-90185, Sweden

**Keywords:** Diet, Cancer, Macronutrients, Carbohydrate intake, Protein intake, Fat intake, Cohort study

## Abstract

**Background:**

Although carbohydrate reduction of varying degrees is a popular and controversial dietary trend, potential long-term effects for health, and cancer in specific, are largely unknown.

**Methods:**

We studied a previously established low-carbohydrate, high-protein (LCHP) score in relation to the incidence of cancer and specific cancer types in a population-based cohort in northern Sweden. Participants were 62,582 men and women with up to 17.8 years of follow-up (median 9.7), including 3,059 prospective cancer cases. Cox regression analyses were performed for a LCHP score based on the sum of energy-adjusted deciles of carbohydrate (descending) and protein (ascending) intake labeled 1 to 10, with higher scores representing a diet lower in carbohydrates and higher in protein. Important potential confounders were accounted for, and the role of metabolic risk profile, macronutrient quality including saturated fat intake, and adequacy of energy intake reporting was explored.

**Results:**

For the lowest to highest LCHP scores, 2 to 20, carbohydrate intakes ranged from median 60.9 to 38.9% of total energy intake. Both protein (primarily animal sources) and particularly fat (both saturated and unsaturated) intakes increased with increasing LCHP scores. LCHP score was not related to cancer risk, except for a non-dose-dependent, positive association for respiratory tract cancer that was statistically significant in men. The multivariate hazard ratio for medium (9–13) versus low (2–8) LCHP scores was 1.84 (95% confidence interval: 1.05-3.23; p-trend = 0.38). Other analyses were largely consistent with the main results, although LCHP score was associated with colorectal cancer risk inversely in women with high saturated fat intakes, and positively in men with higher LCHP scores based on vegetable protein.

**Conclusion:**

These largely null results provide important information concerning the long-term safety of moderate carbohydrate reduction and consequent increases in protein and, in this cohort, especially fat intakes. In order to determine the effects of stricter carbohydrate restriction, further studies encompassing a wider range of macronutrient intakes are warranted.

## Introduction

In recent years, low-carbohydrate diets have emerged as a controversial and popular means of achieving weight loss and controlling diabetes. In Sweden, extensive positive media support for dietary carbohydrate restriction has occurred over the past 5–7 years [1]. During the same time period, in northern Sweden, a complete reversal of previous reductions in fat intake and cholesterol levels has been reported in the general population [2,3]. Discerning the potential long-term health effects of carbohydrate restriction, not only of stringent low-carbohydrate diets but also of more modest carbohydrate reduction, is thus an important challenge in nutrition research today.

For weight loss, low-carbohydrate diets, both extremely or more modestly reduced in carbohydrate (e.g. E% carbohydrates/protein/fat = 9/28/63 [4], and 44/18/38 [5], respectively) have been found to be at least as effective as traditional low-calorie/low-fat diets over a period of up to two years [5-7]. The results of randomized trials have also tended to support improved metabolic parameters and blood lipids [8-11], but elevated markers of stress and inflammation [11-13] in subjects following a low-carbohydrate diet. These alterations might influence the risk of major chronic diseases such as cardiovascular disease and cancer [11,14]. However, from a long-term perspective, the effects of carbohydrate reduction of varying degrees, and consequent increased consumption of various types of protein and/or fat, for health outcomes, and cancer in specific, are largely unknown.

The results of previous epidemiological studies in general populations have suggested positive or null associations between low-carbohydrate diet scores, particularly scores representing diets higher in foods of animal origin, and all-cause, cardiovascular, and cancer mortality [15-19]. Prospective studies of cardiovascular disease incidence have reported either an increased risk [20], or reduced risk for plant-based [21], carbohydrate-restricted diets. The only previous prospective study to address overall cancer incidence found null associations for both animal- and plant-based low-carbohydrate diets [22]. An increased risk of incident breast cancer has been observed for a dietary pattern characterized by a low intake of bread and fruit juice and a high intake of processed meat, fish, butter, other animal fats, and margarine [23]. In contrast, a plant-based, low-carbohydrate diet has been related to a reduced risk of estrogen-receptor-negative breast cancer [24].

Given the high rates of overweight and obesity worldwide, and the widespread popularity of low-carbohydrate diets, evaluation of the long-term safety of carbohydrate restriction of varying degrees is crucial. The aim of the present study was to investigate macronutrient distribution, in particular a previously established low-carbohydrate, high-protein (LCHP) score [16-20], in relation to the risk of incident cancer and specific types of cancer in a large, population-based cohort in northern Sweden.

## Methods

### Study design and cohort

The Västerbotten Intervention Programme (VIP) is an ongoing, population-based, prospective cohort study and health intervention, including residents of the northern Swedish county of Västerbotten turning 40, 50 and 60 years of age. Until 1996, 30 year olds were also included. The VIP protocol, described in detail elsewhere [25,26], includes a health examination, with measurement of a number of potential health risk factors, such as an oral glucose tolerance test, as well as a participant-administered diet and lifestyle questionnaire. For the period assessed in this study, 1990–2007, the average recruitment rate was 59% . The VIP food frequency questionnaire (FFQ) has been validated by 24-hour-recall interviews [27], and by biomarkers in blood samples collected from VIP participants [28,29]. Cancer incidences comparable to those of the general population in Västerbotten indicate a truly population-based cohort [30], and no selection bias of importance has been demonstrated [31].

As of December 31, 2007, when cases of incident cancer were identified for the present study, a total of 82,879 participation occasions (66,001 individuals) had been registered within the VIP cohort. From these we excluded 1,328 participation occasions with missing data for more than 10% of the items in the FFQ and/or portion size, 32 participation occasions with missing height or weight data, 9 participation occasions with a body mass index (BMI) <10, and 6,112 participation occasions with food intake level (FIL) in the lowest 5th percentile or the highest 2.5th percentile (specific to sex and FFQ version and based on the first sampling occasion for subjects with repeated measures), and 12,816 participants with more than one sampling occasion (most recent sampling occasion excluded). The final study population thus included 62,582 participants (31,397 men, 31,185 women).

### Identification of cancer cases

A total of 3,059 incident, prospective cancer cases without previous cancer diagnosis, except non-melanoma skin cancer, were identified through linkage with the regional branch of the national cancer registry, with site-specific cancers defined according to the International Classification of Diseases, ICD-7 [32], as follows: prostate (177), breast (170), colorectum (153, 154.0), respiratory tract (161, 162), urinary tract (181), non-Hodgkin’s lymphoma (200, 202), endometrium (172), malignant melanoma (190), leukemia (204–207), pancreas (157), ovary 175.0), stomach (151), multiple myeloma (203) and renal cell (180.0, 180.9).

### Low-carbohydrate, high-protein score

Dietary intake of macronutrients was calculated from food frequency questionnaires with 9 fixed response alternatives ranging from never to ≥4 times per day and including 84 (years 1990–1996) or 65 (years 1997–2007) food items, as well as photo-based portion-size estimations for meat/fish, potatoes/pasta/rice, and vegetables [26]. The 65-item FFQ was an abbreviated version of the 84-item FFQ, in which some food items had been merged and some deleted as described elsewhere [33] (p 26). All intake variables except ethanol were energy adjusted according to the residual method [34].

Descending deciles, or tenths, of energy-adjusted carbohydrate and ascending deciles of energy-adjusted protein were labeled 1 to 10 and summed to create an LCHP score (2–20 points), a model employed in several previous studies [16-20]. The LCHP score is independent of total energy intake, due to the isocaloric nature of carbohydrate and protein, and it allows separate consideration of the amount and quality of fat consumed. LCHP scores were also categorized into low (2–8 points), medium (9–13 points) and high (14–20 points), in order to approximate equally sized groups.

### Statistical analyses

Differences in baseline characteristics of the study subjects according to LCHP score category were determined by the Kruskal-Wallis test. Spearman’s correlation coefficients were determined between LCHP score and intakes of fat and saturated fat and sex-specific analyses were done. Hazard ratios (HR) and 95% confidence intervals (CI) for overall cancer incidence and for all types of cancer with at least 50 cases were calculated by Cox proportional hazard regression models. HR are presented for medium and high versus low LCHP scores or per 1-point increase in LCHP score. p-trend were calculated per 1-point increase in LCHP score. Age and BMI deviated from the proportional hazard assumption according to Schoenfeld’s test. Age was thus examined in 10-year intervals, included as strata in the crude and multivariate models, and BMI was dichotomized according to obesity (BMI ≥30 kg/m^2^).

Of an extensive list of potential confounding variables, only saturated fat intake altered any HR for LCHP by more than 10% when included in a bivariate model. The final multivariate model included age (10-year intervals, strata), obesity (BMI ≥ 30 kg/m^2^, yes/no), sedentary lifestyle (no physical activity in exercise clothes, yes/no), education (lack of postsecondary, yes/no), current smoking (yes/no), and intake of alcohol (g/day), saturated fat (energy-adjusted residual), and total energy intake (Kcal/day), all selected for their theoretical importance. Missing data, present only for some categorical covariates, i.e. education, N = 377 (0.6%), sedentary lifestyle, N = 1,751 (2.8%), smoking, N = 706 (1.1%), were treated as dummy variables.

Subgroup analyses were conducted for metabolic risk profile, defined as at least one of, versus none of, obesity, diabetes or impaired glucose tolerance. Diabetes was defined as fasting plasma glucose ≥7.0 mmol/l and/or post-load plasma glucose ≥12.2 mmol/l, and impaired glucose tolerance was defined as fasting plasma glucose ≥6.1 mmol/l and/or post-load plasma glucose ≥8.9 mmol/l. Subgroups based on saturated fat intake (energy adjusted and stratified at the median) and energy reporting (adequate versus inadequate, according to the Goldberg cut-off, modified as described in our previous report [19]) were also examined. The subgroup analyses were limited to overall cancer incidence and cancer of the prostate, breast and colorectum, which were the most common sites. Heterogeneity was tested by Chi-square analysis. A sub-analysis was also performed for the time period prior to the shift in macronutrient intake in the VIP population [2] (follow-up to December 31, 2002). All tests were two-sided, and p-values <0.05 were considered statistically significant.

### Ethical considerations

The study was approved by the Regional Ethical Review Board of Northern Sweden (dossier number 07-165 M). All study subjects provided written informed consent, and the study was conducted in accordance with the Declaration of Helsinki.

## Results

Follow-up times ranged from 1 day to 17.9 years, median 9.7 years. Macronutrient intakes for the lowest to highest LCHP scores (2–20 points) ranged from median 60.9 to 38.9% of total energy intake for carbohydrate, 11.3 to 18.9% for protein, and 26.7 to 41.5% for fat. Relationships between baseline characteristics and LCHP score are presented in Table 1. High LCHP scores were associated with younger age (not apparent in medians due to sampling at 10-year age intervals) and higher BMI, prevalence of current smokers, sedentary lifestyle (women only) and alcohol intake. Lack of postsecondary education was more common in men with low LCHP scores and in women with high scores. LCHP scores were positively related to intake of animal protein, but negatively related to plant protein. For carbohydrate and fat, associations were consistent in sucrose and whole grain and saturated and unsaturated fat, respectively. Spearman correlation coefficients for LCHP score and energy-adjusted fat, saturated fat and unsaturated fat intakes were 0.51, 0.45, and 0.46, respectively.

**Table 1:** Baseline characteristics of Västerbotten Intervention Programme participants according to low-carbohydrate, high-protein (LCHP) score

	***n***	**Low**^**1**^**, 2-8**	***n***	**Medium**^**1**^**, 9-13**	***n***	**High**^**1**^**, 14-20**	**p**^**2**^
**Men**							
Age at recruitment, *years*	9,811	50 (40–60)	12,909	50 (40–51)	8,677	40 (40–50)	≤0.001
Follow-up time, *years*	9,811	9.4 (6.4-12.7)	12,909	9.6 (6.1-12.8)	8,677	9.6 (5.2-13.1)	0.206
BMI, *kg*/*m*^*2*^	9,811	25.4 (23.7-27.5)	12,909	25.6 (23.8-27.8)	8,677	26.1 (24.1-28.4)	≤0.001
Current smokers	9,672	1,579 (16.3)	12,728	2,218 (17.4)	8,546	1,672 (19.6)	≤0.001
No postsecondary education	9,753	7,811 (80.1)	12,844	9,957 (77.5)	8,626	6,587 (76.4)	≤0.001
Sedentary lifestyle^3^	9,551	6,633 (69.4)	12,599	8,731 (69.3)	8,371	5,860 (70.0)	0.540
Energy intake, *kcal*/*day*	9,811	2,002 (1,672-2,397)	12,909	2,001 (1,667-2,410)	8,677	2,004 (1,672-2,415)	0.475
Ethanol intake, *g/day*	9,811	4.3 (1.1-7.5)	12,909	4.9 (1.8-8.3)	8,677	5.3 (2.2-8.6)	≤0.001
Carbohydrate intake,% *of energy*	9,811	53.5 (50.7-56.6)	12,909	48.1 (44.7-51.0)	8,677	43.6 (40.6-46.2)	≤0.001
Whole grain intake,% *of energy*	9,811	16.5 (12.2-30.0)	12,909	15.3 (11.1-19.7)	8,677	13.3 (9.4-17.3)	≤0.001
Sucrose intake,% *of energy*	9,811	8.3 (6.3-10.8)	12,909	6.2 (4.6-8.0)	8,677	4.8 (3.5-6.3)	≤0.001
Protein intake,% *of energy*	9,811	12.8 (11.9-13.7)	12,909	14.4 (13.3-15.4)	8,677	16.2 (15.3-17.3)	≤0.001
Animal protein intake,% *of energy*	9,811	8.3 (7.3-9.1)	12,909	10.2 (9.3-11.0)	8,677	12.4 (11.5-13.6)	≤0.001
Plant protein intake,% *of energy*	9,811	4.5 (3.9-5.2)	12,909	4.1 (3.5-4.7)	8,677	3.7 (3.2-4.2)	≤0.001
Fat intake,% *of energy*	9,811	32.7 (29.4-36.0)	12,909	36.7 (32.9-41.1)	8,677	39.4 (36.4-42.7)	≤0.001
Saturated fat intake,% *of energy*	9,811	13.5 (11.7-15.4)	12,909	15.4 (13.4-17.7)	8,677	16.6 (14.9-18.5)	≤0.001
Unsaturated fat intake,% of energy	9,811	18.9 (17.0-20.9)	12,909	21.1 (18.8-23.7)	8,677	22.6 (20.7-24.8)	≤0.001
**Women**							
Age at recruitment, *years*	9,985	50 (40–60)	12,430	50 (40–50)	8,770	50 (40–50)	≤0.001
Follow-up time, *years*	9,985	9.7 (6.7-12.8)	12,430	9.9 (6.6-13.0)	8,770	9.9 (5.9-13.2)	0.206
BMI, *kg/m*^*2*^	9,985	24.4 (22.3-27.2)	12,430	24.4 (22.2-27.2)	8,770	24.7(22.4-27.8)	≤0.001
Current smokers	9,903	1,661 (16.8)	12,319	2,325 (18.9)	8,708	2,152 (24.7)	≤0.001
No postsecondary education	9,913	6,827 (68.9)	12,357	8,612 (69.7)	8,712	6,189 (71.0)	0.005
Sedentary lifestyle^3^	9,749	6,419 (65.8)	12,116	8,121 (67.0)	8,445	5,884 (69.7)	≤0.001
Energy intake, *kcal/day*	9,985	1,509 (1,273-1,787)	12,430	1,531 (1,294-1,815)	8,770	1,510 (1,268-1,806)	≤0.001
Ethanol intake, *g/day*	9,985	1.8 (0.1-3.4)	12,430	1.9 (0.2-3.8)	8,770	2.0 (0.2-4.0)	≤0.001
Protein intake,% *of energy*	9,985	13.4 (12.4-14.2)	12,430	14.9 (13.9-15.8)	8,770	16.7 (15.8-17.8)	≤0.001
Animal protein intake,% *of energy*	9,985	8.4 (7.5-9.3)	12,430	10.3 (9.5-11.1)	8,770	12.6 (11.6-13.7)	≤0.001
Plant protein intake,% *of energy*	9,985	4.9 (4.3-5.5)	12,430	4.5 (3.9-5.0)	8,770	4.1 (3.6-4.6)	≤0.001
Carbohydrate intake,% *of energy*	9,985	56.4 (53.8-59.3)	12,430	51.3 (48.3-53.8)	8,770	47.0 (44.2-49.4)	≤0.001
Whole grain intake,% *of energy*	9,985	18.4 (13.5-23.6)	12,430	16.7 (12.3-21.4)	8,770	14.4 (10.3-18.9)	≤0.001
Sucrose intake,% *of energy*	9,985	7.9 (6.2-10.0)	12,430	6.4 (5.0-7.9)	8,770	5.2 (4.0-6.6)	≤0.001
Fat intake,% *of energy*	9,985	29.1 (25.9-32.2)	12,430	33.0 (29.4-37.1)	8,770	35.5 (32.7-38.7)	≤0.001
Saturated fat intake,% *of energy*	9,985	11.8 (10.2-13.5)	12,430	13.7 (11.9-15.8)	8,770	14.9 (13.4-16.7)	≤0.001
Unsaturated fat intake,% of energy	9,985	17.1 (15.3-19.0)	12,430	19.1 (17.1-21.5)	8,770	20.4 (18.6-22.4)	≤0.001

^1^ LCHP scores were calculated separately for FFQ version and sex, and categorized into roughly equally sized groups: low (2–8 points), medium (9–13 points) and high (14–20 points). Values are medians (25th, 75th percentiles) or frequencies (percents).

^2^ p-values were determined by the Kruskal-Wallis test.

^3^ Defined as no regular physical activity in exercise clothes.

There were no statistically significant associations between LCHP score and any cancer, with the exception of an increased risk of respiratory tract cancer for medium LCHP scores in men (multivariate HR for medium versus low LCHP scores 1.84; 95% CI 1.05-3.23; p-trend = 0.38) (Table 2). HR for high versus low LCHP scores for respiratory tract cancer were above one in both men and women, but not statistically significant.

**Table 2:** Associations between low-carbohydrate, high-protein (LCHP) score and incident all-cause and site-specific cancer in Västerbotten Intervention Programme participants

**Cancer type**	**Sex**	**LCHP score**^**1**^	***n***	**Model 1**^**2,3**^		**Model 2**^**2,4**^	
			**Cases**	**HR (95% CI)**	**p*-*trend**^**5**^	**HR (95% CI)**	**p-trend**^**5**^
All cancer sites	Men	low	545	1		1	
*n* = 3,059		medium	650	1.12 (1.00-1.25)		1.10 (0.97-1.23)	
		high	327	1.01 (0.88-1.16)	0.678	0.97 (0.83-1.12)	0.973
	Women	low	525	1		1	
		medium	596	1.00 (0.89-1.13)		1.00 (0.89-1.13)	
		high	416	1.01 (0.89-1.15)	0.776	1.00 (0.86-1.15)	0.777
Prostate cancer	Men	low	256	1		1	
*n* = 657		medium	266	1.02 (0.86-1.21)		1.01 (0.85-1.21)	
		high	135	0.98 (0.79-1.21)	0.671	0.97 (0.78-1.22)	0.588
Breast cancer	Women	low	195	1		1	
*n* = 581		medium	232	1.03 (0.85-1.24)		1.04 (0.85-1.28)	
		high	154	0.96 (0.78-1.19)	0.761	1.00 (0.79-1.27)	0.924
<49 y at diagnosis	Women	low	30	1		1	
*n* = 104		medium	49	1.15 (0.73-1.81)		1.26 (0.77-2.06)	
		high	25	0.85 (0.50-1.45)	0.948	1.04 (0.57-1.89)	0.343
>55 y at diagnosis	Women	low	73	1		1	
*n* = 184		medium	71	1.03 (0.74-1.42)		1.07 (0.76-1.50)	
		high	40	0.96 (0.65-1.41)	0.975	1.02 (0.67-1.55)	0.707
Colorectum	Men	low	66	1		1	
*n* = 329		medium	75	1.08 (0.78-1.51)		1.02 (0.72-1.44)	
		high	43	1.12 (0.76-1.65)	0.949	1.00 (0.66-1.52)	0.511
	Women	low	53	1		1	
		medium	58	1.02 (0.70-1.48)		0.99 (0.67-1.47)	
		high	34	0.88 (0.57-1.36)	0.625	0.83 (0.52-1.34)	0.459
Respiratory tract	Men	low	19	1		1	
*n* = 143		medium	42	2.10 (1.22-3.61)		1.84 (1.05-3.23)	
		high	18	1.64 (0.86-3.14)	0.044	1.24 (0.62-2.47)	0.381
	Women	low	18	1		1	
		medium	27	1.38 (0.76-2.51)		1.42 (0.76-2.66)	
		high	19	1.39 (0.72-2.65)	0.261	1.37 (0.67-2.82)	0.328
Urinary tract	Both sexes	low	40	1		1	
*n* = 116		medium	47	1.11 (0.73-1.69)		1.11 (0.72-1.73)	
		high	29	1.11 (0.69-1.81)	0.591	1.15 (0.68-1.94)	0.552
Non-Hodgkins lymphoma	Both sexes	low	44	1		1	
*n* = 111		medium	40	0.83 (0.54-1.28)		0.92 (0.59-1.44)	
		high	27	0.90 (0.56-1.46)	0.902	1.10 (0.65-1.88)	0.400
Malignant melanoma	Both sexes	low	34	1		1	
*n* = 105		medium	50	1.21 (0.78-1.87)		1.22 (0.77-1.93)	
		high	21	0.76 (0.44-1.31)	0.475	0.76 (0.42-1.37)	0.509
Endometrium	Women	low	30	1		1	
*n* = 103		medium	41	1.25 (0.78-2.01)		1.35 (0.83-2.21)	
		high	32	1.42 (0.86-2.34)	0.268	1.60 (0.92-2.79)	0.161
Ovary	Women	low	24	1		1	
*n* = 72		medium	28	1.03 (0.59-1.78)		1.01 (0.57-1.79)	
		high	20	1.07 (0.59-1.94)	0.710	0.99 (0.51-1.92)	0.927
Leukemia	Both sexes	low	25	1		1	
*n* = 70		medium	23	0.82 (0.46-1.44)		0.78 (0.43-1.40)	
		high	22	1.20 (0.67-2.14)	0.476	1.14 (0.60-2.15)	0.601
Pancreas	Both sexes	low	25	1		1	
*n* = 70		medium	28	1.03 (0.60-1.76)		0.88 (0.50-1.55)	
		high	17	0.99 (0.53-1.84)	0.771	0.77 (0.39-1.50)	0.584
Stomach	Both sexes	low	24	1		1	
*n* = 69		medium	33	1.27 (0.75-2.16)		1.35 (0.78-2.35)	
		high	12	0.74 (0.37-1.49)	0.301	0.84 (0.40-1.79)	0.526
Multiple myeloma	Both sexes	low	23	1		1	
*n* = 63		medium	29	1.13 (0.66-1.96)		0.94 (0.53-1.68)	
		high	11	0.68 (0.33-1.40)	0.692	0.51 (0.24-1.10)	0.211
Renal cell	Both sexes	low	21	1		1	
*n* = 50		medium	21	0.92 (0.50-1.69)		0.89 (0.47-1.68)	
		high	8	0.56 (0.25-1.27)	0.174	0.54 (0.23-1.30)	0.162

^1^ LCHP scores were calculated separately for FFQ version and sex, and categorized into roughly equally sized groups: low (2–8 points), medium (9–13 points) and high (14–20 points).

^2^ Hazard ratios were determined by Cox regression analyses.

^3^ Including age strata.

^4^ Further adjusted for obesity, sedentary lifestyle, lack of postsecondary education, current smoking, and intake of energy, alcohol, and saturated fat.

^5^ p-trend were calculated per 1-point increase in LCHP score.

Subgroup analyses based on metabolic risk profile, saturated fat intake, and energy reporting [19], had no material effects on results (Table 3). The only statistically significant finding was an inverse association between LCHP score and colorectal cancer risk in women with high saturated fat intakes (multivariate HR for a 1-point increase in LCHP score 0.92; 95% CI 0.87-0.98; p = 0.013; p-heterogeneity = 0.003). Constructing LCHP scores in which whole grain or sucrose replaced total carbohydrates, and in which vegetable or animal protein replaced total protein intake (data not shown), also did not differ from the main findings, except a statistically significant increased risk of colorectal cancer in men with higher LCHP scores based on vegetable protein (multivariate HR for a 1-point increase in LCHP score 1.07; 95% CI 1.01-1.14; p = 0.029; p-heterogeneity = 0.016).

**Table 3:** Associations between low-carbohydrate, high-protein (LCHP) score and incident all-cause and site-specific cancer in subgroups of participants in the Västerbotten Intervention Programme based on metabolic risk profile, saturated fat intake, and energy reporting

**Cancer type**	**Sex**	***n *Cases**	**HR**^**1 **^**(95% CI)**	**HR**^**1 **^**(95% CI)**	**p-heterogeneity**^***2***^
**Metabolic risk profile**^**3**^		**low/high**	**Low**	**High**	
All cancer	Men	1,251/371	1.00 (0.98-1.02)	0.99 (0.95-1.02)	0.668
	Women	1,182/355	1.00 (0.98-1.02)	1.00 (0.97-1.03)	1.000
Prostate cancer	Men	549/108	1.00 (0.97-1.02)	0.98 (0.93 -1.03)	0.513
Breast cancer	Women	468/113	1.00 (0.97-1.02)	1.02 (0.97-1.07)	0.509
Colorectal cancer	Men	142/42	0.98 (0.93-1.02)	1.02 (0.94-1.11)	0.419
	Women	110/35	0.97 (0.92-1.02)	1.04 (0.95-1.14)	0.193
**Saturated fat intake**^**4**^		**low/high**	**Low**	**High**	
All cancer	Men	817/705	1.00 (0.99-1.02)	1.00 (0.98-1.01)	1.000
	Women	845/692	1.01 (0.99-1.02)	1.00 (0.98-1.02)	0.493
Prostate cancer	Men	375/282	1.01 (0.98-1.03)	0.99 (0.96-1.02)	0.363
Breast cancer	Women	320/261	1.01 (0.98-1.04)	0.99 (0.96-1.02)	0.363
Colorectal cancer	Men	89/95	1.00 (0.95-1.06)	0.97 (0.92-1.02)	0.418
	Women	83/62	1.03 (0.98-1.09)	0.92 (0.87-0.98)	0.003
**Energy reporting**^**5**^		**adequate/inadequate**	**adequate**	**inadequate**	
All cancer	Men	548/846	1.00 (0.97-1.02)	1.00 (0.98-1.02)	
	Women	440/996	1.00 (0.98-1.03)	1.00 (0.98-1.01)	1.000
Prostate cancer	Men	250/353	1.00 (0.97-1.03)	1.00 (0.97-1.03)	1.000
Breast cancer	Women	192/360	0.98 (0.95-1.02)	1.01 (0.98-1.04)	1.000
Colorectal cancer	Men	59/108	1.01 (0.94-1.08)	1.00 (0.94-1.04)	0.172
	Women	33/103	1.03 (0.94-1.12)	0.96 (0.92-1.01)	0.837

^1^ Hazard ratios per 1-point increase in LCHP score, determined by Cox regression and adjusted for age strata, obesity, sedentary lifestyle, lack of postsecondary education, current smoking, and intake of energy, alcohol, and saturated fat. Saturated fat intake was not included as a covariate in the subgroup analysis based on saturated fat.

^2^ Comparison of subgroup HR by Chi-square test.

^3^ Metabolic risk profile defined as having at least one of (high), versus none of (low), obesity, diabetes or impaired glucose tolerance.

^4^ Stratified at sex-specific medians.

^5^ Defined according to the Goldberg cut-off, modified as described previously [19].

In analyses restricted to the time period up to and including December 31, 2002, there was a tendency towards a positive association between high LCHP scores and overall cancer risk in both men (multivariate HR for high versus low LCHP scores 1.25; 95% CI 0.86-1.80; p-trend = 0.093) and women, (multivariate HR for high versus low LCHP scores 1.39; 95% CI 0.98-1.96; p-trend = 0.020) (Table 4). For prostate, breast and colorectal cancers no significant associations were found.

**Table 4:** Associations between low-carbohydrate, high-protein (LCHP) score and incident all-cause and site-specific cancer in Västerbotten Intervention Programme participants in a subgroup with reduced follow-up until 2002

**Cancer type**	**Sex**	**LCHP score**^**1**^	***n *Cases**	**Model 1**^**2,3 **^**HR (95% CI)**	**p*-*trend**^**5**^	**Model 2**^**2,4 **^**HR (95% CI)**	**p-trend**^**5**^
All cancer sites	Men	low	94	1		1	
*n* = 531		medium	118	1.20 (0.91-1.58)		1.35 (1.01-1.79)	
		high	55	1.05 (0.75-1.46)	0.599	1.25 (0.86-1.80)	0.093
	Women	low	82	1		1	
		medium	101	1.10 (0.81-1.47)		1.15 (0.84-1.56)	
		high	81	1.29 (0.95-1.76)	0.049	1.39 (0.98-1.96)	0.020
Prostate cancer	Men	low	47	1		1	
*n* = 106		medium	40	0.86 (0.56-1.32)		0.98 (0.63-1.51)	
		high	19	0.80 (0.47-1.38)	0.261	1.01 (0.57-1.80)	0.871
Breast cancer	Women	low	29	1		1	
*n* = 91		medium	33	0.98 (0.59-1.62)		1.05 (0.62-1.78)	
		high	29	1.24 (0.74-2.09)	0.210	1.38 (0.77-2.46)	0.100
Colorectum	All	low	17	1		1	
*n* = 57		medium	24	1.38 (0.74-2.58)		1.48 (0.77-2.81)	
		high	16	1.58 (0.79-3.13)	0.320	1.76 (0.83-3.73)	0.245

^1^ LCHP scores were calculated separately for FFQ version and sex, and categorized into roughly equally sized groups: low (2–8 points), medium (9–13 points) and high (14–20 points).

^2^ Hazard ratios were determined by Cox regression analyses.

^3^ Including age strata.

^4^ Further adjusted for obesity, sedentary lifestyle, lack of postsecondary education, current smoking, and intake of energy, alcohol, and saturated fat.

^5^ p-trend were calculated per 1-point increase in LCHP score.

## Discussion

In this large population-based cohort study with a follow-up period of up to 17.9 years, a diet moderately low in carbohydrates and moderately high in protein was largely unrelated to overall and site-specific cancer incidence, regardless of the quantity and quality of fat intake.

The one previous prospective study to report results for overall cancer incidence, from the Iowa Women’s Health Study, reported inverse risk relationships for isocaloric substitution of either animal or vegetable protein for carbohydrates [22]. However, the results were attenuated to null in multivariate analyses. Associations reported for overall cancer mortality have also been null, non-statistically significant, or unstable [15,17,19,22]. Taken together, the evidence to date does not support a role for moderate carbohydrate reduction in determining the overall risk of cancer.

Increasing LCHP scores were associated with an elevated risk of respiratory tract cancer in both men and women in the present study, but the relationship was not dose dependent and was only statistically significant for medium LCHP scores in men. Although these observations may be due to chance or reflect residual confounding due to smoking, they are consistent with a previous finding for lung cancer mortality [15]. Further study is therefore warranted.

There are several mechanisms by which a carbohydrate-reduced diet could influence carcinogenesis, through specific food items or components, such as red and processed meat for example [35], or through effects on energy metabolism and body composition [36-39]. In analyses considering macronutrient quality and metabolic profile at baseline, two statistically significant results were observed, an inverse association between LCHP score and colorectal cancer risk in women with high saturated fat intakes, and an increased risk of colorectal cancer in men with higher LCHP scores based on vegetable protein. These findings do not support the hypothesis that high animal protein intake increases the risk for these cancer types. Previously, we have reported a null association between LCHP score and colorectal cancer mortality [19], and a positive association has been noted for an animal-based, low-carbohydrate score and colorectal cancer mortality [15]. The latter finding is more consistent with the current understanding of the role of diet in colorectal cancer. For example, there is convincing evidence that a high consumption of protein sources such as red and processed meat is associated with increased colorectal cancer risk [35]. Furthermore, in a controlled trial, a LCHP weight-loss diet has been observed to reduce fecal cancer-protective metabolites and increase hazardous metabolites, which could increase the risk of colon cancer [40].

The limited variability in macronutrient distribution in the study population may have prevented the detection of associations with cancer risk. In particular, the role of stricter carbohydrate restriction could not be assessed. This is an issue common to both the present and previous studies [15,17,19,22]. Interindividual differences, such as gene-nutrient interactions and epigenetics, both emerging research fields [36], may also complicate the relationship between macronutrient distribution and cancer risk. Furthermore, carbohydrate restriction might have different roles in different stages of tumorigenesis, making it difficult to detect overall effects on incidence. For example, putative mechanisms for a role for carbohydrate in the progression from premalignant lesion to cancer diagnosis include metabolic reprogramming of cancer cells resulting in increased glycolysis and glucose requirements, the so-called Warburg effect, as well as the stimulatory effect of insulin-like growth factor on proliferating cells [37,41].

In northern Sweden, a rapid decline in fat intake and hypercholesterolemia occurred between the years 1986–1992 [42], attributed in part to the cardiovascular disease prevention activities of the VIP [42]. Today fat intake has reached the peak levels of the 1980’s, and carbohydrate intake is decreasing [2]. LCHP scores have increased in VIP participants with repeated samples 10 years apart [19]. Furthermore, blood cholesterol concentrations in the northern Swedish population are increasing, despite increasing use of cholesterol-lowering drugs [3]. These temporal changes may have attenuated our results, as indicated by the positive association between a high LCHP score and overall cancer incidence in the sub-analysis restricted to the time period 1990–2002, when LCHP score was relatively stable in the VIP population. In the present study, roughly equal amounts of saturated and unsaturated fat replaced most of the carbohydrate reduction in subjects with high LCHP scores, and the excess protein consumed by subjects with high LCHP scores was primarily of animal origin. In Sweden, extensive positive media focus for carbohydrate-restricted diets in recent years has largely promoted fat, often animal fat, rather than protein, as the substitute for carbohydrates [1,43]. The general enthusiasm for carbohydrate reduction, and the apparent preference for animal-based replacement foods in Sweden, thus underscores the importance of evaluating potential long-term implications for health.

The main strengths of this study were the large, population-based cohort, the extensive data available, such as an oral glucose tolerance test and BMI measured by health professionals, and the long, essentially complete, follow-up. In addition, the prospective study design reduced the risk of recall bias and reverse causation. We used an established LCHP score, which has been employed in previous studies [16-18]. The LCHP score does not include fat intake. However, unlike macronutrient scores that incorporate fat, the LCHP score is independent of total energy intake. It is also simple, both to calculate and interpret, and it allowed separate consideration of the amount and quality of fat consumed. Food frequency questionnaires like the one employed is this study have inherent weaknesses, such as being a relatively inexact tool for the measurement of total nutrient and energy intake, but they are generally adequate for ranking and are an accepted and practical tool for large-scale epidemiological studies. Although several potential confounders were accounted for, residual confounding due to factors not measured (such as food items not included in the FFQ) or not adequately estimated (such as history of tobacco use) was likely present. Since we consider this study to be exploratory, the results were not corrected for multiple testing. The risk of chance findings should therefore be acknowledged. Numbers of cases were also low for some cancer types and in some subgroup analyses.

## Conclusion

In conclusion, the results of this population-based, cohort study do not support an important role for a diet moderately low in carbohydrates and moderately high in protein, regardless of the quantity and quality of fat consumed, in determining the overall, long-term risk of cancer, although a possible increased risk of respiratory cancer was observed and a tendency of an increased general cancer risk over shorter time. Given the current widespread popularity of carbohydrate-restricted diets, and the limited data concerning potential long-term health effects of carbohydrate reduction and consequent increases in protein and/or fat intakes, these findings are important. In order to evaluate the role of more stringent carbohydrate restriction, investigations encompassing a wider range of macronutrient intakes, such as multicenter studies, will be needed.

## Abbreviations

BMI: Body mass index; CI: Confidence interval; FIL: Food intake level; HR: Hazard ratio; FFQ: Food frequency questionnaire; LCHP: Low-carbohydrate high-protein; VIP: The västerbotten intervention programme.

## Competing interests

The authors declare that they have no competing interests.

## Authors’ contribution

LMN, AW, IJ, GH, and BVG designed and conducted the research. LMN analyzed the data. LMN and BVG interpreted the data and drafted the manuscript, and LMN had primary responsibility for the final content. AW, IJ, BL, GH, and PL contributed important scientific content, and all authors revised manuscript drafts and read and approved the final version.
